# EHMTI-0069. The low prevalence of “forehead and facial flushing” and “sensation of fullness in the ear” in Japanese cluster headache patients

**DOI:** 10.1186/1129-2377-15-S1-C24

**Published:** 2014-09-18

**Authors:** N Imai

**Affiliations:** 1 Department of Neurology, Japanese Red Cross Shizuoka Hospital, Shizuoka, Japan

## Background

New ICHD-3 beta criteria for cluster headache added two accompanying autonomic symptoms: “forehead and facial flushing” and “sensation of fullness in the ear”. Previous reports showed that several clinical features of cluster headache were different between Caucasian and East Asian.

## Aim

We evaluated the presence of “forehead and facial flushing” and “sensation of fullness in the ear” in Japan.

## Method

Subjects comprised 23 consecutive cluster headache patients who had been diagnosed with cluster headache according to ICHD-2 (14 males, 9 females; mean age, 41.4±13.8 years; range, 20-71 years). Information was collected by face to face interview.

## Results

All patients were episodic cluster headache. Two patients (9%) had “forehead and facial flushing” and 4 patients (17%) had “sensation of fullness in the ear”. The patients with “forehead and facial flushing” had also “sensation of fullness in the ear”. The prevalence of “forehead and facial flushing” and “sensation of fullness in the ear” in our study were lower than that of previous Caucasian study (20.0% reported “forehead and facial flushing” and 33.3% “sensation of fullness in the ear”).

## Conclusion

The low prevalence of “forehead and facial flushing” and “sensation of fullness in the ear” may be clinical characteristic in Japanese cluster headache patients.

No conflict of interest.

